# TGR5, Not Only a Metabolic Regulator

**DOI:** 10.3389/fphys.2016.00646

**Published:** 2016-12-26

**Authors:** Cong Guo, Wei-Dong Chen, Yan-Dong Wang

**Affiliations:** ^1^State Key Laboratory of Chemical Resource Engineering, College of Life Science and Technology, Beijing University of Chemical TechnologyBeijing, China; ^2^Key Laboratory of Receptors-Mediated Gene Regulation and Drug Discovery, School of Medicine, Henan UniversityKaifeng, China; ^3^Key Laboratory of Molecular Pathology, School of Basic Medical Science, Inner Mongolia Medical UniversityHohhot, China

**Keywords:** TGR5, Gpbar1, GPCR, bile acids, receptor

## Abstract

G-protein-coupled bile acid receptor, Gpbar1 (TGR5), is a member of G-protein-coupled receptor (GPCR) superfamily. High levels of TGR5 mRNA were detected in several tissues such as small intestine, stomach, liver, lung, especially in placenta and spleen. TGR5 is not only the receptor for bile acids, but also the receptor for multiple selective synthetic agonists such as 6α-ethyl-23(S)-methyl-cholic acid (6-EMCA, INT-777) and a series of 4-benzofuranyloxynicotinamde derivatives to regulate different signaling pathways such as nuclear factor κB (NF-κB), AKT, and extracellular signal-regulated kinases (ERK). TGR5, as a metabolic regulator, is involved in energy homeostasis, bile acid homeostasis, as well as glucose metabolism. More recently, our group and others have extended the functions of TGR5 to more than metabolic regulation, which include inflammatory response, cancer and liver regeneration. These findings highlight TGR5 as a potential drug target for different diseases. This review summarizes the basic information of TGR5 and its new functions.

## Introduction

G-protein-coupled receptors (GPCRs) are large family of receptors, playing important roles in multiple pathways (Cvijic et al., 2015). They contain seven transmembrane domains. Upon binding of ligands in the extracellular space, GPCRs transduce the extracellular signal to intracellular downstream cascades through activating multiple effector pathways (Rohrer and Kobilka, 1998). Because of the important functions of GPCR in different cell signaling pathways, they have become attractive targets for treatment of many diseases.

TGR5, as a member of GPCRs, was discovered in 2002 (Maruyama et al., 2002). It was classified as the founder member of the bile acid receptor subclass of GPCRs (Foord et al., 2005). TGR5 gene locates on chromosome position 2q35 in humans. Its open reading frame has 993 base pairs, encoding 330 amino acids. High levels of TGR5 mRNA were detected in several organs such as small intestine, stomach, liver, lung, especially placenta and spleen (Keitel et al., 2007; Tiwari and Maiti, 2009). TGR5 can be activated by bile acids and then it induces cAMP production (Maruyama et al., 2002). As a membrane receptor, TGR5 can be internalized into the cytoplasm in response to its ligands (Kawamata et al., 2003). TGR5 plays important roles in cell signaling pathways such as nuclear factor κB (NF-κB) (Meng et al., 2011), AKT (Kida et al., 2013), and extracellular signal-regulated kinases (ERK) (Masyuk et al., 2013). Its agonists may be potential drugs for treatment of metabolic, inflammation and digestive disorders (Kumar et al., 2012; Broeders et al., 2015).

Activation of TGR5 has shown promise in treating various metabolic diseases such as type 2 diabetes (T2D) and obesity. Its activation also mediates novel effects on inflammation and cancer in different organs. In this review, we summarize the basic properties of TGR5 including its ligands and basic functions. Specifically, we will discuss the new findings about TGR5 in different signaling pathways and diseases.

## The ligands of TGR5

As a plasma membrane-bound GPCR, the endogenous natural agonists of TGR5 are bile acids. Taurolithocholic acid (TLCA), lithocholic acid (LCA), deoxycholic acid (DCA), chenodeoxycholic acid (CDCA), and cholic acid (CA) can dose-dependently induce cAMP production in human TGR5-transfected CHO cells. The rank order of potency (EC50) is TLCA (0.33 μM) >LCA (0.53 μM) >DCA (1.01 μM) >CDCA (4.43 μM) >CA (7.72 μM) (Kawamata et al., 2003) (Table 1). Obacunone, as a limonoid, is found in Citrus. It can dose-dependently stimulate the activity of TGR5 (Horiba et al., 2015). Some other compounds such as linolenic acid (Katsuma et al., 2005) and oleanolic acid (OA) are also identified as weak TGR5 ligands (Sato et al., 2007).

**Table 1:** **Summary of related TGR5 information**.

**Gene**	**GPBAR1, 2q35**	
**Expression in human tissues**	**Placenta, Spleen, Small intestine, Stomach, Liver, Lung, Heart, Skeletal muscle, Kidney, Peripheral blood leukocytes**			
**Natural agonists**		**Name**	**Structures**	**References**
	Primary bile acid	CA	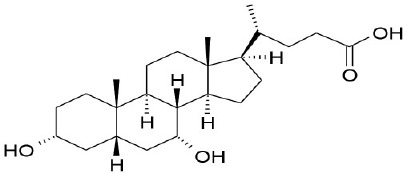	Kawamata et al., 2003
		CDCA	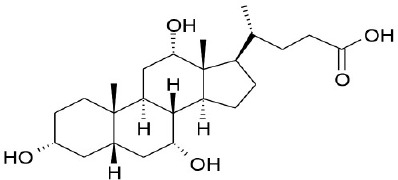	Kawamata et al., 2003
	Secondary bile acid	LCA	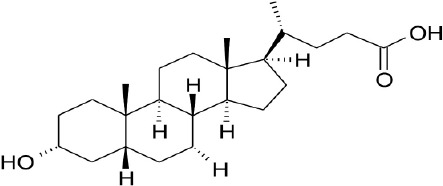	Kawamata et al., 2003
		TLCA	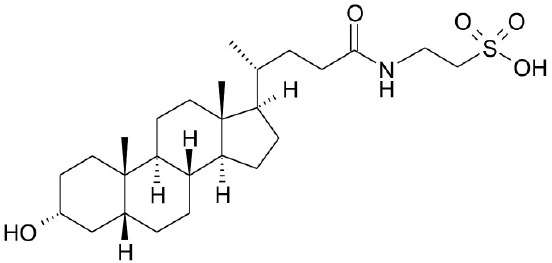	Kawamata et al., 2003
		DCA	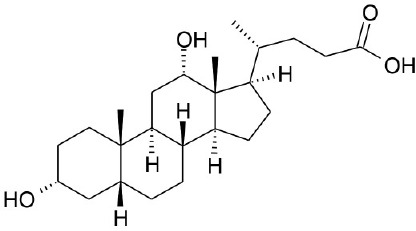	Kawamata et al., 2003
Natural phytochemical agonists		Linolenic acid	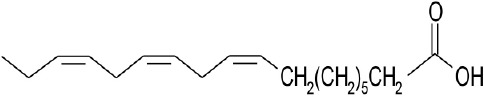	Katsuma et al., 2005
		OA	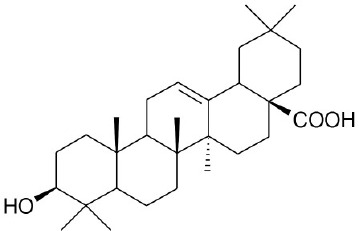	Sato et al., 2007
		Obacunone	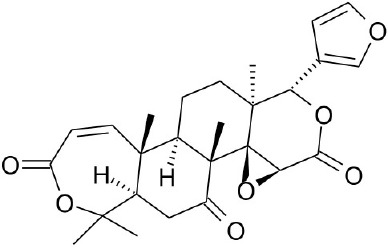	Horiba et al., 2015
Synthetic agonists	INT-777		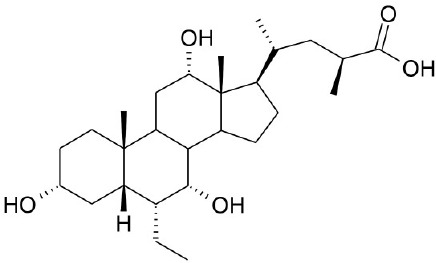	Pellicciari et al., 2009
	TRC210258		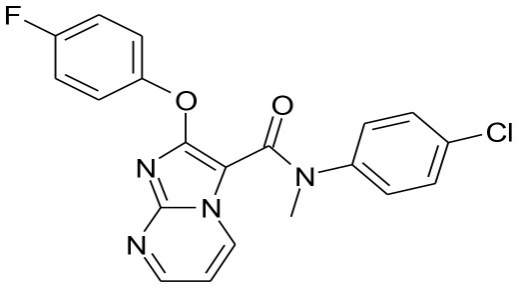	Zambad et al., 2013	
	WB403		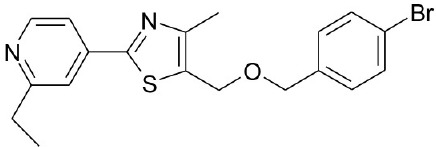	Zheng et al., 2015
**Relevant diseases**			**Agonists**	**References**
	Type 2 diabetes		LCA, DCA, Lionlenic acid, OA, INT-777, WB403	Katsuma et al., 2005; Sato et al., 2007; Perino et al., 2014; Zheng et al., 2015
	Obesity		CA, TCA, DCA, CDCA	Watanabe et al., 2006
	Inflammation		Betulinic acid, 23(S)-mCDCA, TLCA, TLC, CDCA, DCA	Kawamata et al., 2003; Keitel et al., 2008; Wang et al., 2011; Mobraten et al., 2015; Guo et al., 2015b; McMillin et al., 2015
	Gastric cancer		23(S)-mCDCA, GPBARA	Guo et al., 2015a
	Liver regeneration		CA	Péan et al., 2013

CDCA, DCA, LCA, ursodeoxycholic acid (UDCA) are not only the activators of TGR5 but also the activators of farnesoid X receptor (FXR) (Makishima et al., 1999; Wang et al., 2008a,b). In order to find the specific and selective TGR5 ligands, multiple TGR5 agonists were designed and synthesized. Pellicciari et al. reported 23-alkyl-substituted and 6, 23-alkyl-disubstituted derivatives of CDCA are the selective agonists of TGR5 (Pellicciari et al., 2007). 6α-ethyl-23(S)-methyl-cholic acid (6-EMCA, INT-777) had been discovered as a selective, specific agonist for TGR5 (Pellicciari et al., 2009, Table 1). Zhu et al. (2013) designed a new class of potent TGR5 agonists based on 4-phenyl pyridine scaffold. After evaluated *in vitro* and *in vivo*, three compounds showed good effects on activating TGR5. A series of 4-benzofuranyloxynicotinamde derivatives were found to be novel and potent TGR5 agonists (Zou et al., 2014, Table 1). One of them has the highest activity *in vitro* (hTGR5 EC_50_ = 0.28 nM, mTGR5 EC_50_ = 0.92 nM). Zambad et al. (2013) synthesized TRC210258 as a novel TGR5 agonist (Table 1). Zheng et al. found small compound WB403 could activate TGR5 and promote GLP-1 secretion (Zheng et al., 2015).

## TGR5 and cell signaling

### TGR5 and AKT pathway

AKT is a serine/threonine kinase (Faes and Dormond, 2015). It plays important roles in diverse cell processes including differentiation, proliferation, survival, and metabolism (Sasaki and Kuniyasu, 2014). AKT has pleckstrin homology (PH) domain. At the plasma membrane, the interaction between the PH domain of AKT and phosphatidylinositol trisphosphate (PIP3) induces subsequent modifications of AKT at threonine 308. AKT also can be phosphorylated at serine 473. Phosphorylated AKT inhibits pro-apoptotic members of the Bcl-2 family, contributing to cell survival (Sarbassov et al., 2005). In bovine aortic endothelial cells, treatment with TGR5 agonist TLCA enhances AKT phosphorylation and increases NO production (Kida et al., 2013, Figure 1).

**Figure 1:**
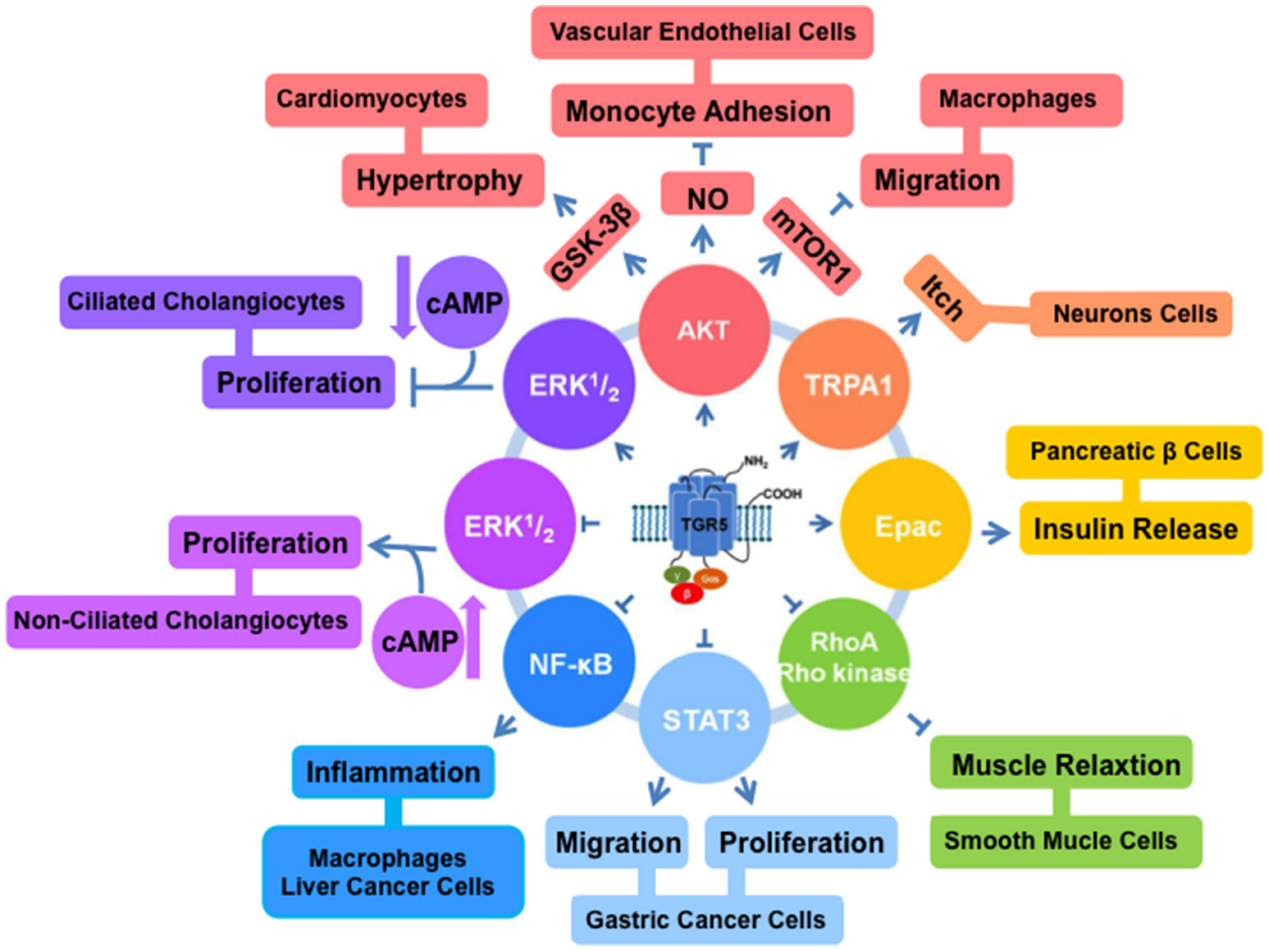
**TGR5 regulates different cell signaling pathways**. TGR5 activates AKT (Kida et al., 2013; Perino et al., 2014), TRPA1 (Lieu et al., 2014), and Epac (Kumar et al., 2012) pathways. And it inhibits NF-κB (Pols et al., 2011; Wang et al., 2011; Yoneno et al., 2013), STAT3 (Guo et al., 2015b), and RhoA/Rho kinase (Rajagopal et al., 2013) pathways. TGR5 has opposite functions in ERK1/2 pathway. In ciliated cholangiocytes, ERK1/2 is activated by TGR5 (Masyuk et al., 2013). But in non-ciliated cholangiocytes, TGR5 activation inhibits ERK1/2 (Masyuk et al., 2013).

Mammalian target of rapamycin (mTOR) is one of the key downstream effectors for the AKT signaling (Covarrubias et al., 2015). mTOR is required for the translation of proteins, which contribute to promoting cell survival and proliferation. TGR5 can reduce chemokine expression via AKT-mTOR pathway in macrophages (Perino et al., 2014). AKT-mTOR pathway can be enhanced through the activation of TGR5. mTOR exists as two complexes mTORC1 and mTORC2. The phosphorylation of AKT and mTORC1 affects the expression of eukaryotic translation initiation factor 4E-binding protein 1 (4E-BP), which is involved in CCAAT-enhancer-binding proteins (C/EBP)β isoform switching. After TGR5 activation, mTORC1 increases the level of phosphorylated 4E-BP and the C/EBPβ isoform liver-inhibitory protein (LIP) expression. The link between TGR5 and AKT-mTOR-LIP reveals a new mechanism by which macrophages contribute to the antidiabetic effects of TGR5 activation (Perino et al., 2014, Figure 1).

### TGR5 and NF-κB pathway

NF-κB is a transcription factor connected with several cellular processes such as inflammation, proliferation, apoptosis and development (Wang et al., 2008c; Meng et al., 2011; Sarode et al., 2015; Papademetrio et al., 2016). NF-κB comprises of five members, RelA (p65), RelB, c-Rel, p50, and p52 (Sun et al., 2013). They are kept inactive in the plasma by binding to family members of IκB including IκBα, IκBβ, IκBγ, BCL3, IκBε, p105, and p100 (DiDonato et al., 2012). Specific IKK kinase regulates IκBα or IκBβ phosphorylation, resulting in activation of NF-κB (Verstrepen and Beyaert, 2014). Two of TGR5 agonists, DCA and LCA, can inhibit tumor necrosis factor-α production in CD14^+^ macrophages (Yoneno et al., 2013). This inhibitory effect is mediated by the phosphorylation of c-Fos to regulate NF-κB p65 activation. Our group identified TGR5 negatively regulated hepatic inflammatory response through antagonizing NF-κB (Wang et al., 2011). We found TGR5 activation suppressed the phosphorylation of IκBα, the translocation of p65, NF-κB DNA binding activity and its transcription activity in HepG2 cells. In the same year, Pols et al. found TGR5 activation by INT-777 decreased nuclear translocation of p65 and phosphorylation of IκBα in macrophages (Pols et al., 2011) (Table 1, Figure 1).

### TGR5 and extracellular signal-regulated kinases (ERK) 1/2 pathway

The kinases ERK1 and ERK2 are members of the mitogen-activated protein kinase family (Pascoli et al., 2014). They are involved in diverse cellular responses such as survival, differentiation, and proliferation (Cheng et al., 2013). In the recent report, Reich et al. (2016) shown that TGR5-selective agonists induced cholangiocyte proliferation through elevation of reactive oxygen species and proto-oncogene, non-receptor tyrosine kinase (cSrc)-mediated epidermal growth factor receptor transactivation and subsequent ERK1/2 phosphorylation in wild type mouse cells. In the ciliated and non-ciliated cholangiocytes, TGR5 activation induces different changes in the levels of cAMP and ERK (Masyuk et al., 2013). TGR5 agonists increase cAMP level and inhibit ERK signaling, resulting in inducing proliferation in non-ciliated cholangiocytes. But in the ciliated cholangiocytes, TGR5 agonists decrease cAMP level and induce ERK signaling, resulting in inhibition of proliferation. The opposite effects of TGR5 agonists are due to the coupling of TGR5 to Gα_i_ protein in ciliated cells and Gα_s_ protein in non-ciliated cells (Masyuk et al., 2013, Figure 1).

### TGR5 and signal transducer and activator of transcription 3 (STAT3) pathway

STAT3 was at first found as a DNA-binding factor in interleukin-6 (IL-6) stimulated hepatocytes. It is an enhancer element in the promoter region of acute-phase genes (Akira et al., 1994). As a transcription factor, STAT3 controls several cellular processes including development, differentiation, immunity, invasion, and metabolism (Kane et al., 2014; Teng et al., 2014; Poli and Camporeale, 2015). It is overexpressed in pathological conditions such as cancer (Yamanaka et al., 1996). Many reports showed STAT3 is activated in various tumor cell lines such as colon, gastric, lung, skin, and breast cancer cells (Levy and Lee, 2002; Yin et al., 2006; Sansone et al., 2007; Yoshimura et al., 2007). Our group found that TGR5 is a suppressor of gastric cancer cell proliferation and migration through antagonizing STAT3 signaling pathway (Guo et al., 2015b). TGR5 activation antagonized STAT3 signaling pathway through suppressing the phosphorylation of STAT3 and its transcription activity induced by lipoplysaccharide (LPS) or IL-6. It suggests that TGR5 antagonizes gastric cancer proliferation and migration at least in part by inhibiting STAT3 signaling. These findings identify TGR5 as an attractive therapeutic target for treatment of gastric cancer (Guo et al., 2015a,b, Figure 1).

### TGR5 and exchange protein directly activated by cAMP (Epac) pathway

Epac is a member of guanine nucleotide exchange factor family and an essential cAMP effector (Gloerich and Bos, 2010). It has multiple binding factors, and is involved in several cellular events (Breckler et al., 2011). In pancreatic β cells, the activation of TGR5 by OA and INT-777 selectively activates Gα_s_. And then the levels of intracellular cAMP and Ca^2+^ will be increased. Epac but not protein kinase A (PKA) can be activated by 8-pCT-2′-O-Me-cAMP, a cAMP analog, and stimulates phosphoinositide (PI) hydrolysis. As the result of the effect, insulin releases from pancreatic β cells (Kumar et al., 2012). In enteroendocrine cells, TGR5 ligand OA can also stimulate Gα_s_ and cAMP formation, and activate Epac increasing PI hydrolysis, glucagon-likepeptide1 (GLP-1) and Peptide YY (PYY) release (Figure 1).

## TGR5 and different diseases

### TGR5 and T2D

Diabetes is one of the fastest deadly growing diseases in the world. T2D is the most common type of diabetes (Zarrinpar and Loomba, 2012). The development of T2D is commonly related to obesity, hypertension, and dyslipidemia (Goedecke and Micklesfield, 2014; Maki and Phillips, 2015). These latter complications promote the development of cardiovascular disease (Johnston et al., 2014). And they are the most common mortality linked to T2D. T2D is classically described as a heterogeneous group of disorders, characterized by a decline in insulin-producing pancreatic β cells, an increase in peripheral insulin resistance, an increase in hepatic glucose production, or a combination of all the factors (Alejandro et al., 2015). Therapies for T2D are made based on reducing hepatic glucose production, increasing insulin secretion, and improving insulin sensitivity (Zarrinpar and Loomba, 2012).

Several studies show the importance of bile acids in glucose homeostasis. Bile acids can improve glycemic control (Zarrinpar and Loomba, 2012). TGR5 as a receptor of bile acids has effect on the regulation of glucose metabolism. In 2005, the study of Katsuma et al. shown the activation of TGR5 could promote GLP-1 secretion in a murine enteroendocrine cell line STC-1 (Katsuma et al., 2005). GLP-1, as the incretin hormone, has the incretin effect, which is the augmentation of insulin secretion after oral administration of glucose. So GLP-1 plays an important role in T2D (Sonne et al., 2014). The secretion of GLP-1 is dose-dependent. The overexpression of TGR5 enhances the level of cAMP and GLP-1 secretion. It suggests that TGR5 induces GLP-1 secretion via intracellular cAMP production (Katsuma et al., 2005). This study aroused the interest of many groups in exploring potential treatment of T2D through the management of glucose homeostasis by activating TGR5. In 2007, OA isolated from olive leaves was found as a natural TGR5 agonist. It decreased plasma glucose and insulin via the activation of TGR5 (Sato et al., 2007). Recent years, it is found that TGR5 induces differential translation of the C/EBPβ isoform LIP by AKT-mTOR pathway in macrophages. And the activation of TGR5 can alter adipose tissue macrophage (ATM) function and improve insulin action. So TGR5 activation in macrophages may prevent insulin resistance and treat T2D (Perino et al., 2014, Table 1). In 2015, a small compound WB403 was identified as a TGR5 agonist. It was tested in the different mouse models of T2D for glycemic control. As a result, TGR5 could be activated by WB403 to improve glucose tolerance, decrease fasting blood glucose and the glycosylated hemoglobin A1c (HbA1c) in T2D mice (Zheng et al., 2015). In the new reports, Kumar et al. (2016) shown that TGR5 induced GLP-1 release from pancreatic α cells via an Epac-mediated PKA-independent mechanism. Agarwal et al. (2016) also shown the important roles of TGR5 in T2D. All of these studies indicate the important functions of TGR5 in T2D treatment.

### TGR5 and obesity

Obesity becomes great threat to public health in the world. The energy intake exceeds expenditure, resulting in obesity (Nalliah et al., 2016). It is now known that brown adipose tissue (BAT) dissipates energy as heat by thermogenesis (Chen et al., 2011). In human BAT, the mitochondria are powerful generators of heat. It metabolizes fat, protecting people from obesity. Because of the key role of BAT in energy burning, increasing BAT amount could be used for treatment of obesity. The administration of bile acids to mice can increase energy expenditure in BAT. This effect is dependent on activation of TGR5, but not FXR (Chen et al., 2011). TGR5 activation increases the level of cAMP-dependent thyroid hormone-activating enzyme, type 2 iodothyronine deiodinase (D2). D2 is one of major thermogenic protein. It can convert thyroxine (T4) into active tri-iodothyronine (T3) in BAT. Bile acid treatment in BAT and human skeletal muscle cells increases D2 activity, oxygen consumption and extracellular acidification rate (Watanabe et al., 2006). In the recent years, different groups also show that the new roles of TGR5 in obesity (Chen et al., 2015; Donepudi et al., 2016; Pierre et al., 2016; Wang et al., 2016). For example, Wang et al. (2016) reported TGR5 inhibited kidney disease in obesity and diabetes through inducing mitochondrial biogenesis and preventing renal oxidative stress and lipid accumulation. These reports suggest that TGR5 agonists may be the potential drugs for treating obesity.

### TGR5 and inflammation

Inflammation is one of the responses of the organism to harmful stimuli, such as pathogens, damaged cells, or irritants (Wang et al., 2008c; Meng et al., 2011). Chronic inflammation is increasingly recognized as an important component of tumorigenesis and metabolic diseases (Coussens and Werb, 2002). Therefore, the precise control of inflammation is essential for the prevention of chronic inflammatory disorders, as well as for inhibiting the exacerbation or progression of diseases, including many types of cancers (Shacter and Weitzman, 2002; Wang et al., 2011).

Our group found the activation of TGR5 could inhibit inflammation in liver and stomach (Wang et al., 2011; Guo et al., 2015a). In liver, TGR5 inhibits the expression of inflammatory mediators in response to NF-κB activation induced by LPS in wild-type (WT), but not TGR5^−/−^ mice (Wang et al., 2011). Yang et al. (2016) reported that during ischemia/reperfusion injury TGR5 inhibited inflammatory response through suppression of the Toll-like receptor 4 (TLR4)-NF-κB pathway. TGR5 activation can also suppress LPS-induced production of cytokines in Kupffer cells and TGR5-overexpressed THP-1 cells (Kawamata et al., 2003; Keitel et al., 2008). But in human monocytes, co-triggering of TGR5 and TLR4 enhances the activation of NF-κB and the production of inflammatory cytokines. The two different and simultaneous events associate with the function of human monocytes, contributing to increasing inflammation (Mobraten et al., 2015). Hepatic encephalopathy (HE) can be a major neurological complication of acute and chronic liver failure. It causes neuroinflammation. The activation of TGR5 by betulinic acid decreases neuroinflammation via neuron and microglia paracrine signaling during HE (McMillin et al., 2015, Table 1). Last year, our group found that TGR5 activation also suppresses gastric inflammation (Guo et al., 2015a). Chronic inflammation is connected with various diseases such as liver, colon and gastric cancer (Guo et al., 2015b). TGR5 may be a potential target for treatment of chronic inflammation and related cancer.

### TGR5 and cancer

Gastric cancer is one of the most common cancers in the world. Gastric carcinogenesis is a complex process and easily causes death (Lin et al., 2015). There are few reports about TGR5 and cancer. Our group found that TGR5 activation could suppress gastric cancer cell proliferation and migration via inhibiting STAT3 pathway (Guo et al., 2015b). Han et al. (2014) demonstrated that the aberrant hypermethylation of TGR5 promoter in serum cfDNA might serve as a biomarker for the surveillance of HCC. Hong et al. (2010) found that TGR5 receptor is over-expressed in oesophageal adenocarcinoma tissues and indicated TGR5 may play an important role in oesophageal adenocarcinoma. The functions of TGR5 in other cancers need to be investigated.

### TGR5 and liver regeneration

Normal liver regeneration is important for restoring the liver mass following liver injury. Previous reports indicate that 70% hepatectomy increases BA flux and changes expression of several nuclear receptors and enzymes involved in BA metabolism (Wang et al., 2008a). The reports shown that bile salts are important for liver regeneration following partial hepatectomy through activating FXR and TGR5 (Wang et al., 2008c; Chen et al., 2010; Fan et al., 2015). In TGR5 knockout mice, exacerbated inflammatory response, severe hepatocyte necrosis, prolonged cholestasis, and delayed regeneration was observed after partial hepatectomy (Péan et al., 2013). So TGR5 has a crucial protective role on the liver in case of BA overload after partial hepatectomy through the control of bile hydrophobicity and cytokine secretion (Zou et al., 2014; Jourdainne et al., 2015).

### Other bile acid membrane receptors

Bile acids also activate other two GPCRs sphingosine-1-phosphate receptor 2 (S1PR2) and muscarinic receptor 2 (Chrm2) (Zhou and Hylemon, 2014). Conjugated bile acids activate S1PR2 to regulate inflammation, cancer development and some liver diseases (Kwong et al., 2015). Muscarinic receptors are overexpressed in colon cancer and their activation promotes proliferation, migration and invasion of human colon cancer cells (Raufman et al., 2003, 2011).

## Prospects

TGR5, as an important membrane receptor, is activated by bile acids and multiple compounds. The novel roles of TGR5 in different diseases make it become a new drug target. Further investigation of TGR5 will provide novel insights into the complex mechanism of metabolic diseases and cancer.

## Author contributions

CG wrote the manuscript, WC and YW revised and edited the manuscript.

### Conflict of interest statement

The authors declare that the research was conducted in the absence of any commercial or financial relationships that could be construed as a potential conflict of interest.
